# Nanotechnology and the Treatment of HIV Infection

**DOI:** 10.3390/v4040488

**Published:** 2012-04-10

**Authors:** Raveen Parboosing, Glenn E. M. Maguire, Patrick Govender, Hendrik G. Kruger

**Affiliations:** 1 Department of Virology, National Health Laboratory Service/University of KwaZulu-Natal, c/o Inkosi Albert Luthuli Central Hospital, 5th Floor Laboratory Building, 800 Bellair Road, Mayville, Durban 4091, South Africa; 2 School of Chemistry, University of KwaZulu-Natal, Varsity Drive, Durban 4001, South Africa; Email: Maguireg@ukzn.ac.za (G.E.M. M.); Kruger@ukzn.ac.za (H.G.K.); 3 School of Biochemistry, Genetics and Microbiology, University of KwaZulu-Natal, Durban 4001, South Africa; Email: Govenderpt@ukzn.ac.za (P.G.)

**Keywords:** Nanotechnology, HIV, antiretroviral agents, nanomedicine, nanoparticles

## Abstract

Suboptimal adherence, toxicity, drug resistance and viral reservoirs make the lifelong treatment of HIV infection challenging. The emerging field of nanotechnology may play an important role in addressing these challenges by creating drugs that possess pharmacological advantages arising out of unique phenomena that occur at the “nano” scale. At these dimensions, particles have physicochemical properties that are distinct from those of bulk materials or single molecules or atoms. In this review, basic concepts and terms in nanotechnology are defined, and examples are provided of how nanopharmaceuticals such as nanocrystals, nanocapsules, nanoparticles, solid lipid nanoparticles, nanocarriers, micelles, liposomes and dendrimers have been investigated as potential anti-HIV therapies. Such drugs may, for example, be used to optimize the pharmacological characteristics of known antiretrovirals, deliver anti-HIV nucleic acids into infected cells or achieve targeted delivery of antivirals to the immune system, brain or latent reservoirs. Also, nanopharmaceuticals themselves may possess anti-HIV activity. However several hurdles remain, including toxicity, unwanted biological interactions and the difficulty and cost of large-scale synthesis of nanopharmaceuticals.

## 1. Introduction

Nanotechnology entails the synthesis and manipulation of materials or systems where at least one dimension is in the nanometer range *i.e.*, in the order of billionths (10^−9^) of a meter [1,2,3,4,5,6,7]. Particles in this size range have unique physicochemical properties, which are distinct from those of bulk materials (the macroscopic or microscopic scale) or single atoms or molecules (the atomic scale) [2,4,5,7,8]. When nanosized particles come into contact with biological systems, the nature of the interaction is critically influenced by these physicochemical properties. Furthermore, many biological phenomena, such as immune recognition and passage across biological barriers, are governed by size considerations. Drugs fabricated at the appropriate nanoscale dimensions may therefore have certain physicochemical and biological properties that in turn confer pharmacological advantages when compared to conventional agents. Research in nanotechnology may translate into benefits for HIV infected patients (Table 1), particularly if the challenges associated with HIV and its treatment are addressed.

## 2. HIV Pathogenesis and Treatment: Challenges and Opportunities

### 2.1. Pathogenesis: Viral Reservoirs (See Figure 1)

HIV most often enters the body via mucosal surfaces and is transported by dendritic cells to lymphoid organs, where it is then delivered to activated CD4^+^ T cells [9,10]. Productive infection of CD4^+^T cells leads to viremia and dissemination of the virus to other sites in the body. Untreated HIV infection is usually associated with high plasma viral loads and progressive decline in CD4^+ ^T cells. Antiretroviral drugs inhibit HIV replication, and treatment with Highly Active Antiretroviral Therapy (HAART), with a regimen consisting of at least three drugs, from at least two classes of antiretroviral agents, will suppress plasma viral load to undetectable levels, and lead to recovery of CD4+ T cell counts [10,11]. However, even optimal treatment with HAART is not able to eradicate HIV. Following acute infection, HIV is able to establish reservoirs within tissues that are inaccessible to optimal levels of antiviral drugs (anatomical reservoirs), or within cells where HIV lies latent, thus escaping the action of antivirals (cellular reservoirs) [12]. The reservoir consists of:

- A large number of extracellular virions, trapped on the surface of follicular dendritic cells within lymphoid tissue [9,10,12], and thus protected from antiretroviral drugs [12].
- A pool of latently infected, resting CD4^+^ T cells [12]. HIV persists even in the presence of HAART because it is not replicating; virus can nevertheless be rescued upon activation of the cell [13].
- Cells of the monocyte-macrophage lineage [12,13] (which constitute the reticulo-endothelial system) and which includes the microglial cells of the brain, pulmonary alveolar macrophages of the lung [12] and macrophages within the spleen and lymph nodes [10,12], Antiretrovirals have poor activity in chronically infected macrophages, requiring much higher intracellular concentrations to achieve inhibition [14]. Macrophages are relatively long-lived cells, and HIV has minimal cytopathic effects on them [12,14,15]. Therefore, macrophages are a persistent reservoir of HIV, even in the presence of HAART.
- Tissues, such as the brain [12], where penetration of antiretroviral drugs is suboptimal, thus allowing continuous, low level replication within cells of the macrophage lineage found at these sites [15].

**Figure 1:**
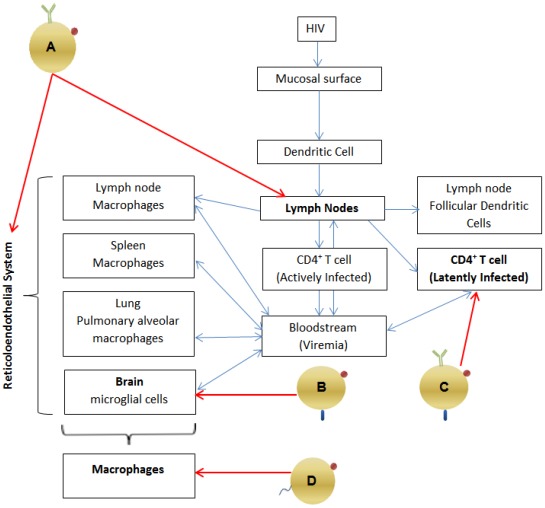
Possible reservoirs of HIV and the potential role of nanotechnology. HIV establishes *anatomical* reservoirs in lymphoid tissue, the reticuloendothelial system and other sites not shown here. Antiretroviral drugs do not penetrate these sites adequately. Macrophages and latently infected CD4^+^ T cells constitute *cellular* reservoirs, because antiretroviral drugs do not achieve satisfactory intracellular concentration within macrophages and antiretrovirals are ineffective against latent virus, respectively [13,14,16,17,18]. Potential means of using nanotechnology to combat viral reservoirs are shown (the relevant sections in the text are indicated): (**A**) Targeted delivery of antiretroviral drugs to the reticuloendothelial system, including lymphatic tissues (Section 8);(**B**) Targeting the brain (Section 9); (**C**) Targeting latently infected CD4^+^ T cells (Section 10); (**D**) Achieving optimal intracellular concentration of antiretroviral drugs within macrophages (the work of Amiji and colleagues is described in the section on nanocarriers, Section 7).

**Table 1:** Examples of how the physical properties of nanoparticles have biological consequences that may benefit HIV therapy [1,2,19,20].

Physical Property	Biological Implications	Potential Benefit for HIV Therapy
Particle size	Particle size affects bioavailability and circulation time [2]. Particles <5–10 nm are removed by renal clearance [21] while those >200 nm are sequestered by the spleen[21]. Particles up to 70 nm can penetrate capillaries [2] while nanocomplexes between 35–120 nm localize in lymph nodes [22,23].	Nanocomplexes containing indinavir were designed so that they were in the size range that allowed localization within lymphatic tissues. Following subcutaneous injection in macaques, the particles drain into the lymphatic system, and because of their size, remain trapped there, rather than entering the bloodstream—this strategy avoids undesirable, excessive peaks in plasma concentration [22,23,24].
	Size determines mechanism of internalization (phagocytosis *versus* endocytosis *versus* pinocytosis) and therefore subcellular localization [21]; depending on their size, particles may be opsonized by plasma proteins, phagocytosed by macrophages and removed by the RES^*^ [21].	Liposomes are phagocytosed by macrophages and deliver drugs such as AZT^+^ [25] and ddI^#^ [26,27], which are carried in their aqueous core, to murine RES^*^.
Large surface area to volume ratio [4]	Dissolution of poorly soluble drugs is greatly dependent on the surface area of the particle. Nanosized particles therefore display enhanced solubility compared to larger particles [2,28,29,30,31].	Engineering drugs in the nanorange, in the form of nanocrystals or nanosuspensions [28,29,30,31,32], for example, allow for clinical development of lead compounds that would not otherwise be considered viable due to poor solubility; Improved solubility enhances bioavailability and dosing of poorly water-soluble antiretroviral drugs, such as rilpivirine [33,34].
Surface charge of particle [2,21]	The cell membrane is negatively charged and repels like-charged molecules. Positively charged nanoparticles may shield such molecules, allowing them to enter the cell [35].	Allows cellular entry of antiretroviral agents, which are negatively charged, such as phosphorylated nucleotide analogues [35] and nucleic acids (See Table 3) [36,37,38,39,40,41,42].
Formation of stable structures which are able to encapsulate drugs [2]	Encapsulation improves solubility and protects against degradation (within the gastrointestinal tract, for example) [43,44].	Polymeric micelles encapsulate efavirenz and improve its solubility [45,46,47,48,49].
		In *in vitro* experiments, a polymeric nanocapsule was used to deliver AZT in its triphosphorylated form directly into the cytoplasm [50,51].
Biofunctionalized nanoparticles, [2,5,52] whereby particles may be functionalized by attachment of bioactive moieties	Nanomedicines are easily tagged by coating them with moieties that bind to biomarkers, thus directing them to cells, tissues or even organelles that exhibit the biomarker [4,21,52].	In animal experiments, liposomes coated with galactose or lectin (“immunoliposomes”) target cells of the RES^*^ that bear receptors for these moieties, and may thus be employed to deliver antiretroviral drugs specifically to these sites [53,54,55,56] (thus decreasing side-effects caused by distribution of drugs to non-specific sites [5]).
	Conjugation to polyethylene glycol (PEGylation) improves solubility and reduces interaction with opsonizing proteins, thus modulating phagocytosis and bioavailability [4].	Sterically stabilized (PEGylated) liposomes and solid lipid nanoparticles, containing ddI^#^ [57] and AZT^+^ [58,59] respectively, result in extended half-lives of these drugs in rodents.
Multifunctionality (combining several beneficial features in a stable construct) [4,7,52]	Currently available antiretrovirals have no effect on latent virus. Nanomedicines may be designed to simultaneously stimulate the replication of latent virus *and* deliver an antiviral to the activated cell [60].	Lipid nanoparticles loaded with bryostatin-2 (which activates primary CD4^+^ T cells) and nelfinavir may be capable of simultaneously activating latent virus and inhibiting viral spread [60].
	The “stealth” properties of polyethylene glycol, which allow drugs to remain longer in the systemic circulation, may be combined with peptides that promote cellular uptake [61].	An HIV TAT**-based peptide (known to have cell penetrating properties), polyethylene glycol and the cell-uptake enhancer, biotin, were conjugated in various combinations and assessed as carriers of saquinavir. The multifunctional bioconjugates had significantly different *in vitro* cellular uptake and anti-HIV potency compared to the prodrug alone [62].
Biomimetic properties [7]	Nanomedicines may “mimic” the properties of biological entities, such as antibodies, receptors, nucleic acids or proteins, by binding to functional sites, such as the active site of an enzyme, thus exerting antiviral effects [7].	Several nanomedicines may have intrinsic antiviral properties (Table 4).
	Synthetic, nanoparticle-based multivalent displays mimic the ubiquitous biological property of multivalency that enhances affinity between naturally occurring molecules (between receptors and ligands, for example) [63].	SDC-1721, a derivative of a known CCR5^****^ antagonist, does not by itself inhibit viral replication. However, when conjugated to gold nanoparticles, at a ratio of 12 SDC-1721 molecules per gold nanoparticle, activity with an IC_50_ of 10 nM was demonstrated in PBMCs ^***^ infected with the CCR5-tropic HIV-1 [63]. Further results are eagerly awaited.

^*^ RES reticuloendothelial system; ^#^ ddI 2', 3'-dideoxyinosine; ^+^ AZT azidothymidine; ^**^ TAT-trans-activator of transcription; ^***^ PBMCs: peripheral blood mononuclear cells; ^****^ CCR5: a chemokine co-receptor used by HIV to enter cells.

Reservoirs are important because they are a source of drug resistant virus (due to ongoing, low level replication in the presence of HAART) and because they make HIV eradication and cure difficult (viral rebound inevitably occurs once HAART is stopped) [16,17,18]. It is therefore essential to explore novel methods to eradicate viral reservoirs. Several sections in this review include synopses of research where the aim is to use nanotechnology to maximize delivery of antiretroviral agents to viral reservoirs:

- Targeting anatomical reservoirs
  ○ reticuloendothelial system (Section 8);
  ○ brain (Section 9);
- Targeting cellular reservoirs
  ○ by optimizing the intracellular concentration of antiretroviral drugs into macrophages (Section 7);
  ○ by activating latent HIV (Section 10).

### 2.2. Antiretroviral Therapy

There are more than 25 anti-retroviral drugs approved for use in HIV-infected individuals [64], from at least six mechanistic classes, which include the nucleoside/nucleotide reverse transcriptase inhibitors, non-nucleoside reverse transcriptase inhibitors, protease inhibitors, fusion inhibitors, CCR5 antagonists and integrase strand transfer inhibitors [11]. However, because they are unable to eradicate viral reservoirs, none of these drugs are curative and lifelong treatment with durable viral suppression is currently the goal of therapy. There are, however, serious challenges to long term suppression of viral replication, which include non-adherence, drug toxicity, drug interactions and the inevitable appearance of drug resistant mutations [65]. 

The convergence of pharmacology and nanotechnology seeks to address some of these issues by creating or enabling some of the following elements:

- Delivery systems that optimize and regulate the tissue distribution and bioavailability of known antiretroviral drugs, thus restricting fluctuating drug levels and toxicity [5].
- Drugs with extended half-life, thus reducing dosing frequency and pill burden [21].
- Targeted drug delivery with an improved side-effect profile [7].
- Delivery systems which reduce drug-drug interactions [66].
- Methods for the delivery of known antivirals delivered by alternative routes (e.g., transdermal delivery) [44].
- Co-delivery of antiretroviral agents, thus improving adherence [7].
- New drugs for the treatment of HIV infection: Newly discovered antiretroviral agents which would otherwise not be of clinical benefit because of solubility issues, may be rendered water-soluble by nanotechnology techniques [7,21].
- Delivery of anti-HIV agents which are currently difficult to deliver e.g., nucleic acids such as siRNA or DNA therapeutics [2]; the delivery vehicle protects the nucleic acid against degradation [5] and reduces immunogenicity [21].
- Drugs with novel mechanisms of action with no cross-resistance to known agents (See Table 4).
- Drugs which target and eradicate viral reservoirs [67].

## 3. The “Nano” Landscape: From Nanomaterials to Nanopharmaceuticals

Nanomaterials are materials engineered in nanometer dimensions to take advantage of novel properties (such as large surface area to volume ratio and quantum effects) that occur at this scale [68]. Nanomaterials are synthesized in either a top-down approach (in which bulk materials or technologies are miniaturized) or a bottom-up approach (where assembly occurs atom by atom, from basic to larger, more complex materials) [1]. Fabrication of materials in the nanoscale results in electronic, magnetic, mechanical and chemical effects that do not occur in bulk materials [1]. These nanoscale effects have been exploited in virtually every field of technology, and include commercial applications in textiles, energy conversion, electronics, cosmetics, lubricants, water purification and computing [69,70]. The study of the interaction of nanomaterials with biological systems is encompassed in the field of “nanobiotechnology” [71], and the related field of nanomedicine seeks to use nanostructured materials to diagnose, treat and prevent human disease [72,73]. 

**Figure 2:**
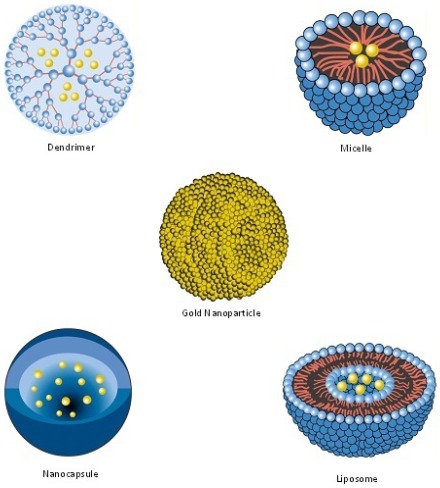
Examples of nanopharmaceuticals and their potential use in HIV infection. **Gold Nanoparticle**: Serves as a scaffold; increases the multivalent display and hence antiviral activity of a CCR5 antagonist [63]. **Nanocarriers**, within which drug molecules, depicted as yellow spheres, are enclosed. **Dendrimers**: Increase the uptake of lamivudine and efavirenz into macrophages [102,106,107]. **Micelles**: Improve the aqueous solubility, oral bioavailability and taste of efavirenz [45,46,47,48,49]. **Nanocapsules**: Increase the uptake of indinavir into the brain [95]. **Liposomes**: Deliver AZT and 2',3' dideoxyinosine preferentially to the RES [25,27,108].

Numerous nanosized pharmaceuticals (“nanopharmaceuticals”) have been investigated for the treatment and prevention of human disease [74] (Table 2 and Figure 2), including HIV. Several reviews have been published that focus on specific aspects or types of anti-HIV nanopharmaceuticals. These include reviews on nanocarriers [44,75], advances in antiretroviral drug delivery [76,77,78,79,80], polymer based nanotechnologies [28,81] or HIV reservoirs [67]. The intention of this review is to provide an overview of all studies in which we identified the application of nanotechnology to HIV treatment (including, *inter alia*, the nanopharmaceuticals listed in Table 2). The reader is referred to several excellent reviews that broadly cover therapeutic as well as preventative facets [82,83,84]. In subsequent sections (which are organized for convenience rather than by convention), we focus on nanopharmaceuticals for the therapy of HIV infection.

## 4. Nanopharmaceuticals and Biological Barriers

Nanopharmaceuticals may enter the body via several routes (intravenous, dermal, subcutaneous, inhalational, intraperitoneal or oral); absorption occurs when they first interact with biological components after which they distribute to various organs in the body. Thereafter, the nanopharmaceuticals may or may not be modified or metabolized and may or may not gain entry into cells, where they may remain indefinitely. During, and following the preceding stages, varying degrees of excretion may occur [85]. The unique properties of each nanopharmaceutical may have beneficial or undesirable effects at each of these steps and furthermore may confer on it either pharmacological activity or the attributes of a suitable nanocarrier. 

During this journey, from the point of administration to the site of antiviral activity, the nanopharmaceutical may encounter numerous biological “barriers” [86,87,88]. These include:

- Enzymatic degradation and poor stability: [87] Nanopharmaceuticals which are stable in experimental or *in vitro* systems may not necessarily be stable when administered to patients.
- Epithelial/endothelial barriers: e.g., the blood-brain barrier [86,88].
- Immunological barriers—opsonization and uptake by the reticuloendothelial system [86]. Opsonization leads to aggregation of nanoparticles and activation of defense mechanisms, including phagocytosis, which removes nanoparticles from the circulation and results in their accumulation in the reticuloendothelial system [89,90].
- Cellular barriers: Inability to traverse the cell membrane [87].
- Extracellular barriers: e.g., inability to penetrate mucin and extracellular matrix [87].
- Intracellular barriers: Entrapment within endosomes, ejection of nanopharaceuticals from the target cell by efflux pumps [88].

Ensuring that a nanopharmaceutical reaches its target site(s) in its active form is a significant challenge in nanotechnology. This challenge may be addressed by optimizing the physicochemical properties of the nanopharmaceutical (charge and size) or by modifying its surface (e.g., biofunctionalization by attachment of ligands or agents that prevent opsonization, facilitate transport across membranes or enable targeting) [61]. Some of the methods are listed below; further examples and details appear in the text and elsewhere [91,92].

*Optimization of the physicochemical properties of a nanoparticle:* Size and charge have an important influence on the stability, biodistribution and efficacy of a nanoparticle (See Table 1) [2,21,61,93]. Depending on its size, a particle may or may not, for example, traverse the endothelial barrier or be sequestered by the spleen, trapped within lymphatic tissues or cleared by the kidney. The size of a nanoparticle also determines the mechanism by which it enters the cell, and where in the cell it localizes. The surface charge of a nanoparticle influences whether or not it traverses the negatively charged cell membrane [35]. Size also significantly influences the opsonization of nanoparticles by plasma proteins [89,90]. 

*Pegylation*: The plasma circulation of particles may be extended by coating them with polyethylene glycol, a process dubbed “pegylation”, which reduces opsonization, phagocytosis and uptake by the RES [90]. Several examples of PEGylated particles, which are often referred to as “stealth” particles, are discussed in sections that follow.

*Overcoming the blood brain barrier*: Penetration of the blood-brain barrier may be achieved by attachment of agents to nanopharmaceuticals that inhibit efflux transporters. Efflux transporters are responsible for poor penetration of certain antivirals into the brain [94,95]. 

*Targeting*: Nanopharmaceuticals may, through active or passive targeting, accumulate preferentially in specific tissues or cells. Passive targeting occurs when nanoparticles (or other therapeutic or diagnostic agents) leak into diseased tissue due to the enhanced permeability of the local vasculature. The increased leakiness of the vasculature may be due to malignancy or inflammation and the therapeutic agent therefore achieves its maximum concentration at the site of disease [96]. In active targeting, ligands attached to a nanopharmaceutical bind specifically to receptors or epitopes that are overexpressed in diseased tissues or cells, thereby causing them to accumulate at the diseased site [96]. 

Targeted delivery to HIV reservoir sites would be of significant benefit because many antiretroviral drugs do not penetrate these sites optimally [97], which contributes not only to viral persistence, but also to the development of drug resistance. Several studies cited in this review employ targeted delivery of antiretroviral agents to reservoir sites such as the brain [98] and reticuloendothelial system [53,54,55,56,99]. Ligand-receptor binding is a method of targeting in which the nanopharmaceutical is tagged with a ligand (such as a peptide, carbohydrate, antibody or antibody fragment), which binds specifically to receptors found on target cells, into which they are internalized. Cells not bearing such receptors are bypassed [54]. These receptors are expressed on the target cells either because the cells are HIV- infected or because such receptors are specifically associated with cell types that are found in reservoir sites. Cells of the reticuloendothelial system, for example, bear HLA-DR determinant of MHC-II [100] and carbohydrate (lectin) receptors [53,54,55,101,102], which may be targeted by anti–HLA-DR monoclonal antibodies and galactose and mannose, respectively. HIV-infected cells express gp120 on their surface, which may be targeted by soluble CD4 ligands [103,104] or fragments of an anti-gp120 monoclonal antibody [105].

The proviso is that targeted HIV therapy cannot be considered without some form of “systemic” (non-targeted) therapy, such as HAART. HIV, far from being a focal disease, is characterized by persistent viremia and affects multiple organs and tissues [12]. Therefore system-wide therapy with HAART (which may not achieve optimal delivery in certain target sites [12]) should be combined with targeted delivery (which may have limited systemic benefit).

**Table 2:** Nanopharmaceuticals defined.

Nanopharmaceutical	Definition
Bucky Ball (Buckminsterfullerene)	A series of carbon atoms arranged in a closed cage structure that resembles a nanosized soccer ball [109].
Dendrimer	Synthetic, nanosized structure made up of multiple branched monomeric units radiating from a central core [110,111].
Liposome	Vesicular nanosized structures made up of one or more phospholipid bilayer membranes surrounding an aqueous core [112].
Micelle	Nanosized structure consisting of a shell and a core (made up of a water-soluble and hydrophobic polymer, respectively) [113,114].
Nanoassembly	Generally, any assembly of hydrophobic and hydrophilic groups that form nanosized aggregates [115]; this article refers to such assemblies brought about by conjugation to squalene^*^ [35,116,117].
Nanoemulsion	Dispersion of immiscible droplets with sizes in the ‘nano’ range [118]
Nanocapsule	A nanosized structure consisting of a shell surrounding a space within which drugs are placed [119].
Nanocarrier	Any nanosized entity used for the controlled and targeted delivery of pharmaceutical agents [2,120]. This is a functional definition; many of the pharmaceuticals listed in this table (including nanoparticles, micelles, dendrimers, liposomes and solid lipid nanoparticles) are defined structurally (in terms of their composition) but may function as, and can be additionally defined as nanocarriers. Further details are provided in Section 7.
Nanocrystal	Drug crystals with a size in the nanometer range [30].
Nanoparticle	Structure with all three dimensions <100 nm [121]. Nanoparticles commonly consist of metals or polymers.
Nanopharmaceutical	Any nanomaterial with therapeutic potential [122].
Quantum dots and rods	Semiconductor nanocrystals [123] having the shape of dots or rods [124].
Solid Lipid Nanoparticle	Particle with a solid lipid matrix and a diameter in the nanometer range [125].

^* ^squalene: A naturally occurring hydrocarbon.

## 5. Nanocrystals

Approximately forty percent of drugs in the drug discovery pipeline that show promising activity are poorly soluble in water [29,30]. Poor solubility leads to erratic bioavailability and suboptimal dosing [29,30] which, in many cases, limits the clinical usefulness and further development of newly discovered agents. The solubility issue of such drugs may be addressed by nanosizing, whereby the drug is reformulated and maintained as nanometer sized crystals (known as nanocrystals), which are then suspended in a liquid (usually water) to form nanosuspensions [31,32]. A stabilizer (surfactant) is usually added to prevent aggregation of the crystals [29,30,31,32]. Since dissolution of poorly soluble drugs is largely dependent on the surface area of the drug particle, and nanoparticles have greater surface area to volume ratios than larger particles, formulating the drug as a nanocrystal may drastically enhance dissolution rates and hence bioavailability [28,29,30,31]. Nanocrystals also have higher drug per volume *i.e.*, higher drug loading, than other nanocarriers (which consist of active drug in addition to the carrier system) [28,31,32]. 

Nanocrystal technology was used to formulate a long-acting, parenteral form of the poorly water–soluble antiretroviral rilpivirine, a next-generation human immunodeficiency virus type 1 (HIV–1) nonnucleoside reverse transcriptase inhibitor [126,127]. The half-life of conventional rilpivirine is 38 hours [128]; in contrast, when rilpivirine nanocrystals (comprising rilpivirine as the free base or its corresponding HCl salt, an aqueous carrier and a hydrophilic surfactant) were injected subcutaneously in dogs, plasma concentrations were sustained for 3–6 months [33,34], providing proof of concept that a nanosuspension of rilpivirine may serve as a long-acting injectable. The benefits of such a formulation in humans would be decreased dosing frequency, improved adherence, reduced impact of food on bioavailability and fewer toxicities (a lower dose of rilpivirine can be administered since first-pass metabolism is bypassed) [33,34]. The feasibility of subcutaneous delivery of antiretroviral agents has been demonstrated in clinical trials with enfuvirtide, an approved antiretroviral agent in current use [129].

Nanocrystals injected into the venous circulation are opsonized by plasma proteins and are phagocytosed rapidly and predominantly by the Kuppfer cells of the liver, which serves as a depot for the accumulation and slow release of the drug. This phenomenon may be an advantage (if reticuloendothelial accumulation and slow release is desired) or a disadvantage (if the drug is toxic to liver cells, and if high plasma levels are required). Depending on the application, nanocrystals may be fabricated to be <100 nm. These so called “smartCrystals” avoid phagocytosis and furthermore, due to their large surface area to volume ratio, undergo rapid dissolution in the bloodstream, resulting in a “bolus” effect post-injection. Alternatively, a “stealth” particle may be formulated, which is a nanocrystal coated with polyethyleneglycol, which prevents opsonization, thus promoting prolonged circulation. A “homing” molecule (which mediates its attachment to the target cell) may additionally be used for targeted delivery of the nanocrystal, which may be preferred to non-specific accumulation in the reticulendothelial system or rapid dissolution in plasma [31]. An example is the targeted delivery of nevirapine nanocrystals to the brain, facilitated by surface modification with albumin [98]. However, the mechanism by which albumin facilitates delivery of nanocrystals through the blood–brain barrier is not clear. Further details of this experiment appear in Section 9, Targeting the Brain.

## 6. Nanoassemblies

Squalene, a steroid precursor, is a biocompatible hydrocarbon that is widely distributed in nature and is commonly used as an excipient in the pharmaceutical industry [130]. When squalene is covalently conjugated to nucleoside analogues, they self-organize in aqueous media to form nanosized structures called “nanoassemblies” [35,116,117]. Nanoassemblies have enhanced pharmacological properties compared to the underivatized parent drug [130]. Nanoassemblies may be created, for example, to allow nucleosides to enter the cell in their prodrug, monophosphorylated form. Nucleoside analogues do not usually enter the cell in this form, due to repulsion between their phosphate group and the cell membrane, both of which are negatively charged. Nucleosides therefore depend on cellular kinases for phosphorylation, which is a rate-limiting step in their activation [35]. Conjugation to squalene facilitates direct entry of monophosphorylated nucleosides into the cytoplasm, because the nanoassemblies shield the negative charge of the phosphate group from the cell membrane [35]. In an example of this novel prodrug delivery strategy, nanoassemblies of squalenoyl dideoxycytidine monophosphate tested in HIV-1 infected peripheral blood mononuclear cells displayed twice the *in vitro* anti-HIV potency of the parent molecule, suggesting that the negatively charged nucleotide analogues efficiently penetrated the cell membrane [35].

In a further application, polyethylene glycol-coated, or “stealth” nanoassemblies, may be used to encapsulate nucleoside analogues and protect them against degradation and rapid elimination from plasma [116].

## 7. Nanocarriers

Nanocarriers are submicron entities (with a diameter typically between 10 and 1,000 nm) used for the controlled delivery of pharmaceutical agents that are encapsulated within, or adsorbed or conjugated onto their surface [2,120]. The therapeutic agent gains pharmacological advantages brought about by the nanometer size range of the complex (which influences passage across cell membranes, uptake by the reticuloendothelial system, dissolution rates, solubility and bioavailability), while its own activity, ideally, remains unaffected [2]. Various polymeric and non-polymeric materials may be used as nanocarriers; these include nanoparticles, micelles, dendrimers, liposomes and solid lipid nanoparticles [2,44,77,78]. 

*Polymeric nanoparticles.* *Polymeric nanoparticles* consist of a polymeric matrix with a therapeutic agent attached onto its surface or encapsulated within its interior [2]. Amiji and colleagues used a nanoparticle carrier system to increase the cellular uptake of the antiretroviral agent, saquinavir into a monocyte/macrophage cell line. In this system, saquinavir was loaded into a poly(ethylene oxide)-modified poly(epsilon-caprolactone) (PEO-PCL) nanoparticulate system. PCL is a synthetic, biocompatible polymer that is highly permeable to many drugs, which makes it suitable as a carrier system, while PEO was used to prevent chain aggregation. Saquinavir uptake by THP-1 cells was significantly higher with saquinavir in the nanoparticle formulation compared to aqueous solution [131].

*Micelles.* Nanocarrier encapsulation may also be exploited to improve solubility and increase oral bioavailability of poorly water-soluble drugs. Polymeric micelles, nanosized structures consisting of a water-soluble polymer that forms the “shell” and a hydrophobic polymer that forms the drug-encapsulating core [113,114], may be used for this purpose. In a series of studies by Sosnik and colleagues, efavirenz was incorporated into the core of amphiphilic linear and branched poly(ethylene oxide)-poly(propylene oxide) block copolymer micelles [45,49]. This significantly improved the aqueous solubility of efavirenz (by about 8400 times), which in turn improved the oral bioavailability of efavirenz in a liquid (pediatric) form, in rats [47,48]. Encapsulation also improves the taste of efavirenz [46], a significant consideration in the pediatric population [45,46,47,48,49]. In other studies, encapsulation with β-cyclodextrin, which has a hydrophobic center and hydrophilic outer surface, increased saquinavir’s solubility 27-fold, improved oral bioavailability in rats ninefold [132] and significantly enhanced the dissolution rate of efavirenz [133].

*Nanocapsules.* A nanocapsule consists of a nanosized shell surrounding a space within which drugs may be placed. A polymeric nanocapsule may be used to carry nucleoside reverse transcriptase inhibitors (NRTIs) in their triphosphorylated form directly into the cytoplasm. NRTIs are active only in this triphosphate form, but phosphate groups are too hydrophilic and do not usually traverse the cell membrane [134]. NRTIs delivered in their conventional non-phosphorylated form therefore require obligatory intracellular phosphorylation by cellular kinases. The inefficiency of cellular kinases may limit the activity or use of some nucleoside analogues. A promising strategy to bypass this metabolic bottleneck involves the use of a nanocapsule to transport azidothymidine-triphosphate (AZT-TP) directly into the cytoplasm. This nanocapsule consists of a poly(*iso*-butylcyanoacrylate) aqueous core which entraps the AZT-TP, and polyethyleneimine which prevents “leakage” of AZT-TP [50,51]. 

*Others.* Antiretrovirals may also be complexed with a variety of other nanocarriers such as dendrimers, liposomes, solid lipid nanoparticles, lipid-drug nanocomplexes, nanoemulsions of essential polyunsaturated fatty acids, cationic emulsomes and low-density lipoproteins. These function as nanocarrier systems, but have additional features, which are discussed in the relevant sections that follow.

## 8. Targeting the Reticulo-Endothelial System (RES)

Liposomes are nanosized vesicular structures made up of one or more phospholipid bilayer membranes surrounding an aqueous core [112]. Liposomes and several other nanocarriers are readily opsonized by plasma proteins, phagocytosed by macrophages and localize to cells of the reticuloendothelial system (RES) [77,135,136]. The RES is a significant reservoir of HIV replication [137]. Hydrophilic drugs encapsulated in liposomes localize in organs rich in macrophages, such as liver, spleen and lungs. In a study using mice infected with the murine AIDS virus, liposomes were used to deliver AZT preferentially to the RES, sparing the bone marrow from AZT and thereby reducing bone marrow toxicity [25]. Similarly, liposome-encapsulated 2',3' dideoxyinosine (ddI) was efficiently localized within the reticuloendothelial system following intravenous bolus injection in female Sprague-Dawley rats. This resulted in reduced systemic clearance and higher plasma drug levels of ddI [27,108]. 

The surface of liposomes may be modified to further increase their targeting specificity. Cells of the reticuloendothelial system bear galactose and lectin receptors; galactosylated [54,55,56] and mannosylated [53,99] liposomes target these receptors, respectively, and have been used to direct AZT, ddI and stavudine to the reticoloendothelial system. Immunoliposomes have the targeting specificity of antibodies on their surface e.g., anti-HLA-DR monoclonal antibodies which target follicular dendritic cells, B cells and macrophages which express the HLA-DR determinant of MHC-II. Such immunoliposomes resulted in enhanced accumulation of indinivar in mice lymph nodes, with area-under-the-curve exceeding that of free drug 126-fold [100]. Liposomes may also be decorated with recombinant soluble CD4 molecules [103,104], the Fab’ fragment of monoclonal antibody F105 [105] or Fab’ fragments of anti-HLA-DR antibody [138], all of which target gp120 on HIV infected cells [103].

Other lipid-containing nanocarriers also target lymphatic tissues. In macaque-based studies, nanocomplexes incorporating indinavir with phosphatidylcholine and cholesterol lipids achieve about 22 times higher concentration of indinavir in lymph nodes compared to plasma, and 10-fold reduction in peak plasma concentrations of indinavir [22,24]. In further studies, nanocarriers composed of disteroyl phosphatidylcholine and methyl polyethylene glycol disteroyl phosphatidylethanolamine achieved sixfold higher indinavir levels in lymph nodes of macaques than other lipid nanocarriers [23]. Furthermore, complexes with surface polyethylene glycol exhibit the highest drug levels in lymph nodes [23]. A possible explanation of the lymph node localization of these complexes is as follows: The diameter of the nanocarrier complex (35 to 120 nm) overlaps that of lymph node drainage (50 to 100 nm), but exceeds that of the endothelial pores (generally up to 12 nm in diameter) [139]. After subcutaneous delivery, the nanocarrier complex therefore enters, distributes and accumulates within the lymphatic system but is not able to traverse the endothelial barrier, and will therefore not readily appear in the bloodstream. The release of indinavir from liposomes is pH-dependent since the lipophilicity of indinavir is pH-dependent. In plasma, at physiological pH, indinavir reduces water–solubility and associates with the lipids, whereas, at lower pH within the endosome, indinavir is water-soluble, dissociates from the lipid, and is released. Indinavir is acid-stable and retains its activity [23].

Several polymeric nanoparticles are also phagocytosed by cells of the reticuloendothelial system. For example, poly-(lactic-co-glycolic acid) nanoparticles containing ritonavir, lopinavir, and efavirenz resulted in intracellular peak levels in peripheral blood mononuclear cells that continued until day 28 compared to free drug, which was eliminated in two days [140]. Similarly, in rats, AZT concentrations were 18 times higher in the reticuloendothelial system if AZT was bound to hexyl-cyanoacrylate nanoparticles compared to unbound AZT [141,142,143]. 

Dendrimers, which are synthetic, polymeric, tree-like structures in which multiple highly-branched monomeric units radiate from a central core [110,111], may likewise be phagocytosed by macrophages when they are conjugated with molecules that are the ligands of receptors on the surface of macrophages. For example, mannosylated fifth-generation poly(propyleneimine) dendrimers may be used to increase the uptake of lamivudine [102] and efavirenz [106] by targeting them to lectin receptors on the surface of macrophages. Tuftsin, a natural macrophage activator tetrapeptide, when conjugated to poly(propyleneimine) dendrimers (TuPPI), increased the cellular uptake of efavirenz 34.5-fold [107]. 

Liposomes coated with PEG (“sterically-stabilized liposomes”) are less readily opsonized, and therefore have longer plasma half-life, significantly improved bioavailability and greater accumulation in lymphatic tissues than non-PEGylated liposomes [144]. In a study in rats, 2',3'-dideoxyinosine encapsulated in sterically stabilized liposomes had extended half-life in plasma compared to conventional liposomes (14.5 *versus *3.9 hours) [57]. The bioavailability of AZT incorporated within solid lipid nanoparticles may also be enhanced by the use of PEG (creating so called “stealth solid lipid nanoparticles” that have modified circulation times) [58,59]. Similarly polymeric nanoparticles, e.g., poly(lactide acid), when coated with PEG, are less efficiently phagocytosed by macrophages. This may be used to modulate the delivery of AZT to the reticuloendothelial system [145].

## 9. Targeting the Brain

Nanomaterials may also be used to achieve targeted delivery of antiretrovirals to the brain. Many antivirals have limited distribution in brain tissue due to the permeability glycoprotein (P-gp) efflux transporter. Certain agents, such as the polymer Pluronic P85 and the excipient Solutol® HS15, block P-gp and therefore improve delivery of antivirals through the blood-brain barrier. The Pluronic block copolymer P85, which probably works by reducing the availability of ATP to P-gp [146], enhanced the *in vivo *efficacy of antiretroviral drugs zidovudine, lamivudine and nelfinavir in a severe combined immunodeficiency (SCID) mouse model of HIV-1 encephalitis (HIVE) [94]. Similarly indinavir, when loaded into Solutol® HS15 nanocapsules, achieved significantly higher tissue *versus *plasma concentration in mice brains compared to administration as a solution (nanocapsule *versus* solution ratio of 1.9 in normal mice and 1.5 in P-gp-deficient mice) [95].

Several lipid containing nanosystems also limit binding to efflux transporters. The accumulation of atazanavir in a human brain microvessel endothelial cell line (hCMEC/D3) was significantly enhanced when encapsulated by solid lipid nanoparticles, suggesting that this is a promising method for delivery of atazanavir across the blood-brain barrier [147]. The maximum concentration and the area-under-the curve values in the brain were respectively five- and threefold higher than the aqueous suspension when saquinavir was delivered by an oil-in-water nanoemulsions made with essential polyunsaturated fatty acid-rich oils [148].

Polybutylcyanoacrylate and methylmethacrylate-sulfopropylmethacrylate nananoparticles also increased the permeability of the blood-brain barrier to several antiretrovirals, but the exact mechanism of action of these polymers is unclear [149,150]. Interestingly, the permeability across endothelial cells in these systems is affected by exposure to electromagnetic interference, possibly because of the effect of the charge of the nanocarrier on permeability [151]. 

Regions of the TAT peptide known as protein transduction domains are able to pass through biological membranes independent of transporters. TAT-conjugated poly(L-lactide) nanoparticles enhanced the uptake of ritonavir in mice brain 800-fold [152].

Finally, targeted delivery across the blood-brain barrier may also be achieved with the use of surface modified nanocrystals. In a study using rats, intravenously administered nevirapine nanocrystals that were surface-modified with serum albumin showed enhanced accumulation in the brain compared to nanocrystals modified with polysaccharide or polyethylene glycol; all forms of the nanocrystal, including the unmodified form, accumulated predominantly in the liver and the spleen [98]. The mechanism of this effect is not clear; further research may reveal a possible method of targeting viral reservoirs in the brain.

## 10. Targeting Latent HIV

In a recent study using human T-cell lines and a humanized mouse model, lipid nanoparticles loaded with bryostatin-2, a protein kinase C activator, were able to activate primary CD4^+^ T cells and stimulate HIV replication in latently infected cells. Furthermore, if the particles are additionally loaded with an antiretroviral such as nelfinavir, the multifunctional nanoparticle will be capable of simultaneously activating latent virus and inhibiting viral spread [60]. This proof-of-concept study suggests the possibility that nanotechnology may provide the “holy grail” of HIV eradication research: A drug that will “flush out” and purge the latent viral reservoir.

## 11. Bio-Functionalized Nanoparticles

**Figure 3:**
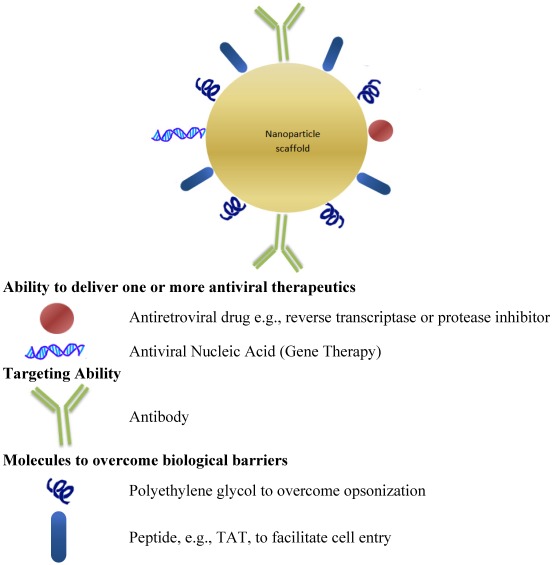
Hypothetical biofunctionalized, multifunctional nanopharmaceutical with the following [52,86,153,154].

Nanopharmaceuticals may be created by conjugating a nanomaterial with a biologically derived or biologically based component such as a nucleic acid, protein, peptide or antibody (See Figure 3). A peptide based on the sequence of the HIV TAT (which is known to have cell-penetrating properties), the polymeric carrier polyethylene glycol and the cell uptake enhancer, biotin, were conjugated in various combinations and assessed as carriers of saquinavir. *In vitro* experiments demonstrated that the multifunctional bioconjugates had significantly different cellular uptake and anti-HIV potency compared to the prodrug alone [62].

Polyethylene glycol (PEG) may be biofunctionalized to improve its ability to carry antiretroviral and other drugs to cellular targets, including macrophages. PEG has useful “stealth” properties [61]: It protects attached drugs from enzymatic degradation, prevents rapid renal clearance and inhibits interaction with cell-surface proteins. This allows PEGylated complexes to remain in the systemic circulation longer. However the restricted interaction of PEG with cell surfaces (the very property which makes it useful) also hinders its use as a drug carrier (since antiretrovirals need to reach their target cell to be effective) [155]. Attachment of a cell-targeting peptide to PEG overcomes this limitation. An example of a cell targeting peptide is *N*-formyl-Met-Leu-Phe (fMLF). fMLF resembles a bacterial protein and is a known chemo-attractant for macrophages, to which it binds avidly; fMLF may be used to functionalizes PEG. Experiments in mice suggest that targeting macrophages using fMLF-modified PEG may be a feasible strategy [155].

## 12. Gene Therapy: Nanocarriers of RNA and DNA

RNA-based therapies are an attractive prospect for the treatment, not only of HIV [83,156,157], but of a wide variety of other infectious and non-infectious diseases [158]. Strategies include ribozymes, antisense RNAs, RNA aptamers, RNA decoys, human ribonuclease P (RNase P), modified small nuclear RNA and small interfering RNAs [156,157]. However RNA delivery to target sites, by vector and non-vector methods, has been plagued by several challenges that restrict the clinical utility of RNA therapeutics. These issues include rapid degradation in physiological conditions, short half-life, poor cellular uptake, subcellular compartmentalization, unwanted interactions with plasma proteins and the immune system, low bioavailability and off-target side-effects [36,37,38,159,160,161,162,163,164,165]. Nucleic acid delivery by vector methods has also posed potential safety concerns due to their oncogenic, inflammatory and immunogenic effects [39].

Numerous methods for non-viral delivery of therapeutic DNA [161] and RNA [40,160,166] have been explored. In particular, nanosized drug carrier systems have been developed [38,41] that may address many of the issues related to nucleic acid delivery: Improved safety (due to biodegradability and rapid clearance by the reticuloendothelial system), targeted delivery and controlled release, improved uptake by overcoming electrostatic repulsion between negatively charged RNA or DNA and cell membranes, improved stability in physiological fluids and protection from degradation [36,37,167]. Several of these approaches have been adopted for the delivery of anti-HIV RNA and DNA therapeutics (Table 3). In general, the nucleic acid is condensed with a cationic reagent via electrostatic interactions. The cationic reagent (which may be a peptide, liposome or dendrimer) protects the nucleic acid against degradation and facilitates cellular uptake by endocytosis [42].

**Table 3:** Potential anti-HIV nucleic acids and their delivery by nanocarriers.

Nanocarrier	Nucleic acid	Target	Effect
Poly(L-lysine), a cationic peptide	antisense oligonucleotide	primer binding site and U5 region of the viral genome	In cell culture, the antisense oligonucleotide covalently linked to poly(L-lysine) inhibited HIV-1 reverse transcriptase mediated elongation of cDNA, in a sequence and dose-dependent manner [168].
Protamine, a cationic peptide	antisense oligonucleotides	tat^***^ mRNA	Protamine bound to the antisense oligonucleotide led to specific inhibition of tat-mediated HIV-1 transactivation in lymphocytes [169].
Quantum rod	siRNA^#^	poly A/TAR (transactivator of the HIV-1 LTR^**^) site	The quantum rod-siRNA nanoplex suppressed HIV-1 viral replication in a THP-1 cell line [170].
Amino-terminated carbosilane dendrimers	siRNA^#^	p24 region of gag or the nef sequence	Dendrimers formed dendriplexes with siRNA^#^, which were delivered to human astrocytes, where they reduced the replication of HIV-1 [171].
Amino-terminated carbosilane dendrimers	siRNA^#^	p24, gag and nef	Dendriplexes were able to transfect the lymphocytic cell line SupT1 and hard-to-transfect HIV-infected peripheral blood mononuclear cells (PBMCs), where they reduced HIV replication [172].
pH-sensitive liposomes	Antisense oligodeoxynucleotide; ribozyme	Rev responsive element and 5'-LTR^**^, respectively	Inhibited virus replication in monocyte-derived macrophages [173]. However, delivery of functional ribozymes by liposomes is relatively inefficient [174].
LFA-1^*^- targeted and stabilized immunoliposome nanoparticles	siRNA^#^	CCR5	siRNA^#^ administered to humanized mice using immunoliposome nanoparticles resulted in selective uptake of siRNA^#^ by T-cells and macrophages and reduction in HIV plasma viral load [175].

^* ^LFA-1: Lymphocyte function-associated antigen-1 integrin, expressed on all leukocytes; ^**^ LTR: Long terminal repeat; ^*** ^tat: Trans-activator of transcription; ^# ^siRNAs: small interfering RNAs.

**Table 4:** Nanopharmaceuticals with potential anti-HIV activity.

Nanopharmaceuticals	Proposed Mechanism of Action	Activity
Silver nanoparticles [176]	The nanoparticles interact preferentially, and in a size-dependent manner [177], with gp120 knobs and prevents CD4-dependent virion binding, fusion, and infectivity [178].	TI^**^ = 8.9 [178]
Phenyldicarboxylic acid and naphthyldisulfonic acid polyanionic dendrimers	Interacts with gp120 and interferes with virus-cell binding.	EC_50_^* ^of 0.1 and 0.3 µg/mL, respectively [179]
One-tailed, long-chain, water-soluble, dendritic tricarboxylato amphiphiles	They most likely act by blocking viral fusion [77].	TI^**^ = 4 and EC_50_^*^ = 110–740 µM [180]
Phosphorus-containing dendrimers bearing Galβ_1_cer^*** ^analogues	Block HIV entry.	IC_50_ values of 1.1 and 0.12 µM, respectively[181]
Polylysine-sulfated cellobiose^## ^glycodendrimer	Electrostatic interaction between negatively charged sulfated oligosaccharide and positively charged gp120 on the surface of HIV [182].	EC_50_^*^ = 3.2 µg/mL [182]
Mannose hyperbranched dendritic polymers	Inhibits binding of HIV gp120 to DC-SIGN^### ^[183].	IC_50 _= 50 µM [183]
Polyamidoamine (PAMAM) dendrimers	Binds to TAR^#^ RNA and prevents its interaction with Tat^**** ^protein [184].	not known
Multivalent glycosphingolipid-derived carbohydrate head groups covalently attached to a dendrimer core	Inhibits interaction between HIV gp120 and glycosphingolipids, which are alternate receptors for HIV-1 on the surface of immune cells [185].	IC_50_ between 0.1 and 7.4 μg/mL [185]
Sialic Acid-Polyamidoamine (PAMAM) Glycodendrimers	Probably bind to and down-regulate CD4 antigen on surface of T-cells [186]	IC_50 _between 1.6 and 5.1 μM [187]
Water soluble dendrimic fullerene	Dendrimers seem to bind to HIV-1 protease.	EC_50_^*^ = 0.22 µM [188]
“Bucky Ball” (C60 fullerene) structures	Computational docking models and kinetic analysis suggest binding of Bucky Ball derivatives to the active site of HIV-1 protease [189].	not known

^* ^EC_50_: Effective concentration 50%, the concentration of drug that achieves half the maximum protective effect; by comparison, the EC_50 _of AZT and lopinavir are 0.186 µM [190] and 19 nM [191] , respectively;^ **^ TI: Therapeutic index, a measure of effectiveness *versus* toxicity, the higher the index, the better the drug; expressed as the ratio of the concentration of the drug at which it is toxic to 50% of the cells *versus* the concentration at which it protects 50% of the cells; by comparison, the equivalent index for AZT is 1027 [191] and that of lopinavir is >1,000 [191];^ ***^Galβ_1_cer: Galactosylceramide; ^#^ TAR: Trans-activation response region; ^**** ^tat: Trans-activator of transcription; ^## ^cellobiose: An oligosaccharide; ^### ^DC-SIGN: Dendritic cell-specific intercellular adhesion molecule (ICAM)-3 grabbing non-integrin, a lectin expressed on the surface of dendritic cells, involved in early stages of HIV infection.

## 13. Nanopharmaceuticals with Potential Antiviral Activity

Nanopharmaceuticals themselves may exert antiviral activity by targeting one or more steps in the replication cycle of HIV. Metal nanoparticles, dendrimers and “Bucky Balls”, for example, bind HIV enzymes or proteins thereby blocking the replication of HIV, possibly by steric hindrance (Table 4). These mechanisms are size-dependent in that some inorganic metals (such as silver), for instance, possess antiviral effect when present in nanometer-sized macromolecules but have limited or no activity in bulk or atomic form [176,177,178]. 

## 14. Limitations

Several challenges in the field of HIV nanotherapeutics should be acknowledged and addressed. Firstly, there are biological challenges. Some nanoparticles are degraded in the gut following oral administration, or fail to penetrate the mucus barrier and are thus minimally absorbed [192]. Others may have unwanted interactions with biological systems (e.g., plasma proteins), which leads to opsonization, uptake by macrophages and reduced plasma half-life [7]. The default response to many nanomedicines may unfortunately be that the body is “programmed” to get rid of them, by phagocytosis and other mechanisms. 

Nanotherapeutics may have unacceptable toxicity. The very properties that make them useful may also lead to undesirable consequences. For example, the larger surface area to volume ratio of certain nanomaterials may exaggerate their toxic effects [5]. Nanoparticles may be absorbed and distributed nonspecifically [7], are usually endocytosed and may have unpredictable intracellular effects, may induce apoptosis and disrupt cell membrane [7], and may generate adverse immunological responses [31]. In addition, they may be too large for renal clearance and if they cannot be degraded within the body, they will accumulate, leading to toxicity [31,193]. Inorganic nanoparticles, in particular, may not be easily degraded or metabolized, and once absorbed will remain in the body for years [192]. 

Traditional methods for assessing cytotoxicity and efficacy may be complicated by the unique features of nanomaterials [4]. Data is lacking on how nanoparticles are metabolically processed [7]. Much research is therefore required in the field of nanotoxicology [7,31].

Secondly, there are technological hurdles. Scaling up is challenging and expensive. Optimization at a laboratory scale is much simpler than at an industrial or commercial level [193]. 

## 15. Perspectives

In this review, diverse applications of nanotechnology have been described: Some have the potential to improve the pharmacological profile of known antiretroviral agents: Nanocrystals to allow formulation of antiretroviral agents as long acting injectables; nanoassemblies and nanocapsules to allow nucleoside reverse transcriptase inhibitors to enter the cell in phosphorylated form; micelles to improve oral bioavailability and taste. Several methods to target viral reservoirs have also been explored: Polymeric nanoparticle to improve uptake of antiretrovirals in macrophages; liposomes, polymeric nanoparticles and dendrimers to target the reticuloendothelial system (with targeting ligands to improve specificity); nanocapsules, nanocrystals and other nanosystems to improve delivery across the blood-brain barrier; a multifunctional nanoparticle to target latent HIV. Several *in vitro* experiments suggest possible methods to facilitate the delivery of therapeutic nucleic acids into cells. Finally, metallic and dendrimer-based nanopharmaceuticals showed potential anti-HIV activity in cell culture. 

However, none of the research described here has proceeded beyond the pre-clinical stage. The success of any nanopharmaceutical depends on at least three criteria [77,82,83], none of which can be satisfactorily or completely assessed without clinical data [28,83]: Firstly, the nanopharmaceutical should exert an antiviral effect, secondly, it should have an acceptable toxicity profile and thirdly, it should be stable and be able to overcome biological barriers. The challenge is that optimizing one criterion may be detrimental to the others, e.g., optimizing efficacy (by, for instance, including an additional therapeutic agent into a multifunctional nanoparticle) may exacerbate toxicity (because such an agent may be toxic) and decrease stability (because the more complex construct is likely to be less stable). Extensive *in vitro* optimization experiments may be necessary to achieve the ideal construct for *in vivo* evaluation. Further research is needed to enhance our understanding of how to design and interpret these *in vitro* experiments. Specific issues regarding the reproducibility of experiments and the interaction with biological systems (such as coating of the nanoparticles with proteins in the cell culture medium) need to be resolved [194]. Assays to assess safety and efficacy need to be standardized and validated [88,153]. Computerized models, that predict or simulate nanopharmaceutical behaviour, need to be developed and optimized [88]. Nevertheless, the eventual success of these efforts will be determined in the clinic rather than the laboratory.

An additional issue that needs to be considered is whether the use of the nanopharmaceutical leads to HIV drug resistance. For example, targeted delivery of an antiretroviral drug, except when it is absolutely specific or accompanied by systemic HAART, will lead to suboptimal doses of the drug in non-targeted tissues, with the potential to select out drug resistant mutations there. Furthermore, most proof of concept studies involving nanocarriers use a single antiretroviral drug, which would effectively select out resistant virus in targeted tissues. It is more appropriate, as in conventional HAART, to combine at least three drugs for use with nanocarriers [28,67,77].

Multifunctionalization, which includes the concept of combining several antiretroviral drugs onto one carrier, is a promising avenue of research in nanotechnology [44]. Further functionalization could involve targeted delivery to specific cells or tissues, improved cellular uptake or the avoidance of opsonization, *etc.* Multifunctionality may be the key property that establishes the superiority of nanopharmaceuticals over conventional agents.

Research that addresses these challenges and opportunities may pave the way for nanotechnology to make a significant impact in the lives of those infected with HIV.
